# Supplementary material for: HIVprotI: an integrated web based platform for prediction and design of HIV proteins inhibitors
Source: J Cheminform. 2018 Mar 9;10:12. doi: 10.1186/s13321-018-0266-y (PMC5845081; doi:10.1186/s13321-018-0266-y)
Supplement: Supplementary file 2 — Additional file 2. Source code of HIVProtI web server. [file 13321_2018_266_MOESM2_ESM.zip › HIVprotI_Source-code/hivprotI/weka-3-6-6/doc/weka/core/matrix/SingularValueDecomposition.html]

SingularValueDecomposition


---


|  |  |  |
| --- | --- | --- |
| **Overview** \| **Package** \| **Class** \| **Tree** \| **Deprecated** \| **Index** \| **Help** \| **Weka's home** | |  |
| **PREV CLASS**   NEXT CLASS | **FRAMES**    **NO FRAMES**     **All Classes** |  |
| SUMMARY: NESTED \| FIELD \| CONSTR \| METHOD | DETAIL: FIELD \| CONSTR \| METHOD |  |


---


## weka.core.matrix Class SingularValueDecomposition

```
java.lang.Object
  weka.core.matrix.SingularValueDecomposition
```

**All Implemented Interfaces:**: java.io.Serializable, RevisionHandler

---

``` public class SingularValueDecomposition extends java.lang.Object implements java.io.Serializable, RevisionHandler ```

Singular Value Decomposition.

For an m-by-n matrix A with m >= n, the singular value decomposition is an
m-by-n orthogonal matrix U, an n-by-n diagonal matrix S, and an n-by-n
orthogonal matrix V so that A = U\*S\*V'.

The singular values, sigma[k] = S[k][k], are ordered so that sigma[0] >=
sigma[1] >= ... >= sigma[n-1].

The singular value decompostion always exists, so the constructor will never
fail. The matrix condition number and the effective numerical rank can be
computed from this decomposition.

Adapted from the JAMA package.

**Version:**
:   $Revision: 1.5 $

**Author:**
:   The Mathworks and NIST, Fracpete (fracpete at waikato dot ac dot nz)

**See Also:**: Serialized Form

---

**Constructor Summary**

`SingularValueDecomposition(Matrix Arg)`
  
          Construct the singular value decomposition


| **Method Summary** | |
| --- | --- |
| `double` | `cond()`             Two norm condition number |
| `java.lang.String` | `getRevision()`             Returns the revision string. |
| `Matrix` | `getS()`             Return the diagonal matrix of singular values |
| `double[]` | `getSingularValues()`             Return the one-dimensional array of singular values |
| `Matrix` | `getU()`             Return the left singular vectors |
| `Matrix` | `getV()`             Return the right singular vectors |
| `double` | `norm2()`             Two norm |
| `int` | `rank()`             Effective numerical matrix rank |

**Methods inherited from class java.lang.Object**

`equals, getClass, hashCode, notify, notifyAll, toString, wait, wait, wait`

**Constructor Detail**

### SingularValueDecomposition

```
public SingularValueDecomposition(Matrix Arg)
```

:   Construct the singular value decomposition

    **Parameters:**: `Arg` - Rectangular matrix


**Method Detail**

### getU

```
public Matrix getU()
```

:   Return the left singular vectors

    :   **Returns:**: U

---


### getV

```
public Matrix getV()
```

:   Return the right singular vectors

    :   **Returns:**: V

---


### getSingularValues

```
public double[] getSingularValues()
```

:   Return the one-dimensional array of singular values

    :   **Returns:**: diagonal of S.

---


### getS

```
public Matrix getS()
```

:   Return the diagonal matrix of singular values

    :   **Returns:**: S

---


### norm2

```
public double norm2()
```

:   Two norm

    :   **Returns:**: max(S)

---


### cond

```
public double cond()
```

:   Two norm condition number

    :   **Returns:**: max(S)/min(S)

---


### rank

```
public int rank()
```

:   Effective numerical matrix rank

    :   **Returns:**: Number of nonnegligible singular values.

---


### getRevision

```
public java.lang.String getRevision()
```

:   Returns the revision string.

    :   **Specified by:**: `getRevision` in interface `RevisionHandler`
    :   **Returns:**: the revision


---


|  |  |  |
| --- | --- | --- |
| **Overview** \| **Package** \| **Class** \| **Tree** \| **Deprecated** \| **Index** \| **Help** \| **Weka's home** | |  |
| **PREV CLASS**   NEXT CLASS | **FRAMES**    **NO FRAMES**     **All Classes** |  |
| SUMMARY: NESTED \| FIELD \| CONSTR \| METHOD | DETAIL: FIELD \| CONSTR \| METHOD |  |


---
